# Tissue Doppler velocity imaging and event timings in neonates: a guide to image acquisition, measurement, interpretation, and reference values

**DOI:** 10.1038/s41390-018-0079-8

**Published:** 2018-08-02

**Authors:** Eirik Nestaas, Ulf Schubert, Willem P. de Boode, Afif El-Khuffash, on behalf of the European Special Interest Group ‘Neonatologist Performed Echocardiography’ (NPE), T Austin, K Bohlin, M. C. Bravo, C. R. Breatnach, M Breindahl, E Dempsey, A. M. Groves, S Gupta, B Horsberg Eriksen, P. T. Levy, P. J. McNamara, Z Molnar, S. R. Rogerson, C. C. Roehr, M Savoia, C. E. Schwarz, A Sehgal, Y Singh, M. G. Slieker, C Tissot, R van der Lee, D van Laere, B van Overmeire, L van Wyk

**Affiliations:** 1 Institute of Clinical Medicine, Faculty of Medicine, University of Oslo, Oslo, Norway; 2 Department of Cardiology and Center for Cardiological Innovation, Oslo University Hospital, Rikshospitalet, Oslo, Norway; 3 Department of Paediatrics, Vestfold Hospital Trust, Tønsberg, Norway; 4 Department of Clinical Science, Intervention and Technology, Karolinska Institutet, Stockholm, Sweden; 5 Department of Neonatology, Radboud University Medical Center, Radboud Institute for Health Sciences, Amalia Children’s Hospital, Nijmegen, The Netherlands; 6 Department of Neonatology, The Rotunda Hospital, Dublin, Ireland; 7 Department of Pediatrics, The Royal College of Surgeons in Ireland, Dublin, Ireland; 8 Department of Neonatology, Rosie Hospital, Cambridge University Hospitals NHS Foundation Trust, Cambridge, United Kingdom; 9 Department of Neonatology, Karolinska University Hospital, Karolinska Institutet, Stockholm, Sweden; 10 Department of Neonatology, La Paz University Hospital, Madrid, Spain; 11 Department of Neonatology, The Rotunda Hospital, Dublin, Ireland; 12 Karolinska University Hospital, Karolinska Institutet, Stockholm, Sweden; 13 INFANT Centre, Cork University Maternity Hospital, University College Cork, Cork, Ireland; 14 Division of Newborn Medicine, Mount Sinai Kravis Children’s Hospital, New York, NY USA; 15 University Hospital of North Tees, Durham University, Stockton-on-Tees, United Kingdom; 16 Department of Pediatrics, Møre and Romsdal Hospital Trust, Ålesund, Norway; 17 Department of Pediatrics, Washington University School of Medicine, Saint Louis, MO USA; 18 Department of Pediatrics, Goryeb Children’s Hospital, Morristown, NJ USA; 19 Departments of Pediatrics and Physiology, University of Toronto, Toronto, ON Canada; 20 John Radcliffe Hospital, Oxford, United Kingdom; 21 The Royal Women′s Hospital, Parkville, VIC Australia; 22 Department of Paediatrics, University of Oxford, John Radcliffe Hospital, Oxford, United Kingdom; 23 Azienda Ospedaliero-Universitaria S. Maria della Misericordia, Udine, Italy; 24 Department of Neonatology, University Children’s Hospital of Tübingen, Tübingen, Germany; 25 Department of Pediatrics, Monash University, Melbourne, Australia; 26 Addenbrooke′s Hospital, Cambridge University Hospitals NHS Foundation Trust, Cambridge, United Kingdom; 27 Department of Paediatric Cardiology, Radboudumc Amalia Children’s Hospital, Nijmegen, The Netherlands; 28 Department of Pediatrics, Clinique des Grangettes, Chêne Bougeries, Switzerland; 29 Department of Neonatology, Radboud university medical center, Radboud Institute for Health Sciences, Amalia Children’s Hospital, Nijmegen, The Netherlands; 30 Department of Pediatrics, Antwerp University Hospital UZA, Edegem, Belgium; 31 Department of Neonatology, University Hospital Brussels, Brussels, Belgium; 32 Department of Paediatrics & Child Health, University of Stellenbosch, Cape Town, South Africa

## Abstract

Neonatologists can use echocardiography for real-time assessment of the hemodynamic state of neonates to support clinical decision-making. There is a large body of evidence showing the shortcomings of conventional echocardiographic indices in neonates. Newer imaging modalities have evolved. Tissue Doppler imaging is a new technique that can provide measurements of myocardial movement and timing of myocardial events and may overcome some of the shortcomings of conventional techniques. The high time resolution and its ability to assess left and right cardiac function make tissue Doppler a favorable technique for assessing heart function in neonates. The aim of this review is to provide an up-to-date overview of tissue Doppler techniques for the assessment of cardiac function in the neonatal context, with focus on measurements from the atrioventricular (AV) plane. We discuss basic concepts, protocol for assessment, feasibility, and limitations, and we report reference values and give examples of its use in neonates.

## Introduction

An increasing number of neonatologists have been trained to use echocardiography for the assessment of the hemodynamic status of infants to support clinical decision-making. Conventional echocardiographic indices such as shortening fraction (SF) and ejection fraction (EF) have several shortcomings.^1–7^ Tissue Doppler imaging (TDI) is an emerging technique that can provide measurements of movement of the myocardium and timing of myocardial events in the cardiac cycle.

There is an increasing body of literature on the use of TDI in neonates. The aim of this review is to provide an up-to-date overview of TDI techniques for assessment of cardiac function in the neonatal context, with a focus on measurements from the atrioventricular (AV) plane. We discuss basic concepts of TDI, protocol for assessment, feasibility and limitations, provide examples of its use in neonates and suggest directions for research and further exploration.

## Why TDI in neonates?

The main task of the cardiovascular system is to provide the body with a sufficient supply of oxygenated blood to maintain adequate cellular metabolism. Cardiac output is determined by myocardial contractility, heart rate, preload, and afterload. No echocardiographic index of cardiac function can assess the contractility of the myocardium per se. Ambient loading conditions have an impact on all cardiac function indices.^8^ Conventional measures of cardiac function generally assess changes in cavity dimensions and blood velocities rather than provide direct information on myocardial characteristics. Those include SF, EF and pulsed-wave Doppler measurements of blood flow. Several studies have demonstrated the relative lack of sensitivity of those measures in detecting myocardial dysfunction.^2–7^ EF can be calculated from SF,^9^ but as calculation of EF from SF is not feasible in hearts with asynchronous contraction and distorted geometry, newer guidelines recommend assessing the EF by Simpson biplane method.^1^

As most studies in neonates have used TDI to assess longitudinal motion of the AV-plane viewed from the apical position, this will be the acquisition modality discussed in this paper. When assuming a stationary position of the apex relative to the probe during the cardiac cycle, movement of the AV-plane will be the result of the average function of the myocardial wall between the apex and the AV-plane. TDI has the advantage of high time resolution (high frame-rate) and ability to assess both left and right cardiac function. Unlike deformation imaging by strain and strain rate indices, assessment of AV-plane motion does not offer regional myocardial function assessment. The lack of spatial resolution is, however, probably less relevant in neonates than in adults, in whom regional functional disturbances due to ischemia and scars are important aspects of cardiac function evaluation.

## Principles and basic concepts of TDI

The algorithms for assessing velocities of the myocardium are similar to the algorithms for assessing velocities of the blood. The ultrasound signals from blood and myocardium differ in two aspects; the signals reflected from myocardium are stronger (higher amplitudes) and the velocities are lower. The analysis software separates the signals from tissue and blood by these properties.

**Fig. 1:**
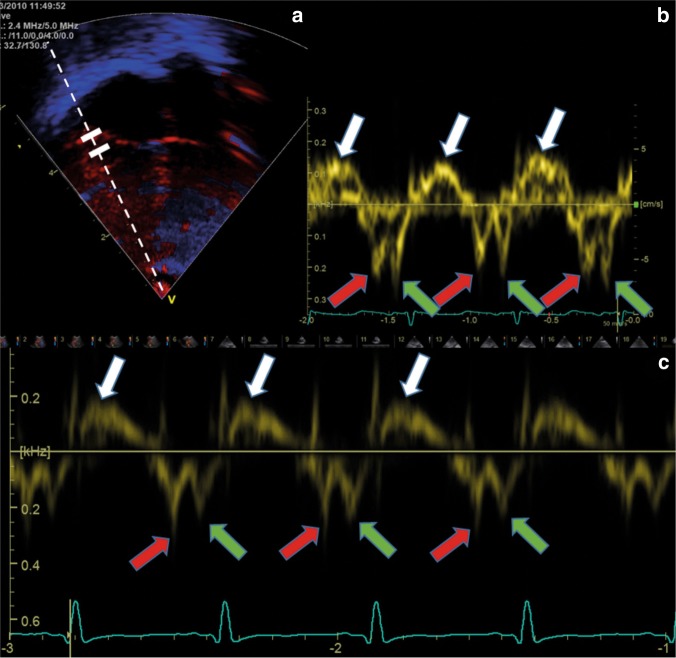
Pulsed-wave tissue Doppler velocity measurement from a term neonate, from the right lateral atrioventricular plane from the apical four-chamber view. The ultrasound system records the velocities from the area between the two calipers (the two white solid lines in the upper left panel). The dotted line denotes the direction of the ultrasound beams assessing the velocities. (Upper left panel) **a** Color-coded tissue Doppler image. (Upper right panel) **b** Spectral velocity time curve of three heart cycles, with relatively high gain setting. The upper panels (**a** and **b**) show a relatively poor image quality (blurred 2D image (**a**) and a broad spectral velocity–time curve (**b**). The lower panel **c** shows a velocity curve with narrower spectral curve, indicating better quality. *X*-axis: time. *Y*-axis: tissue velocity from the sample area. White arrows at the peak systolic velocities (s′), red arrows at the early diastolic velocities (e′), and green arrows at the peak velocity in atrial systole (a′)

**Fig. 2:**
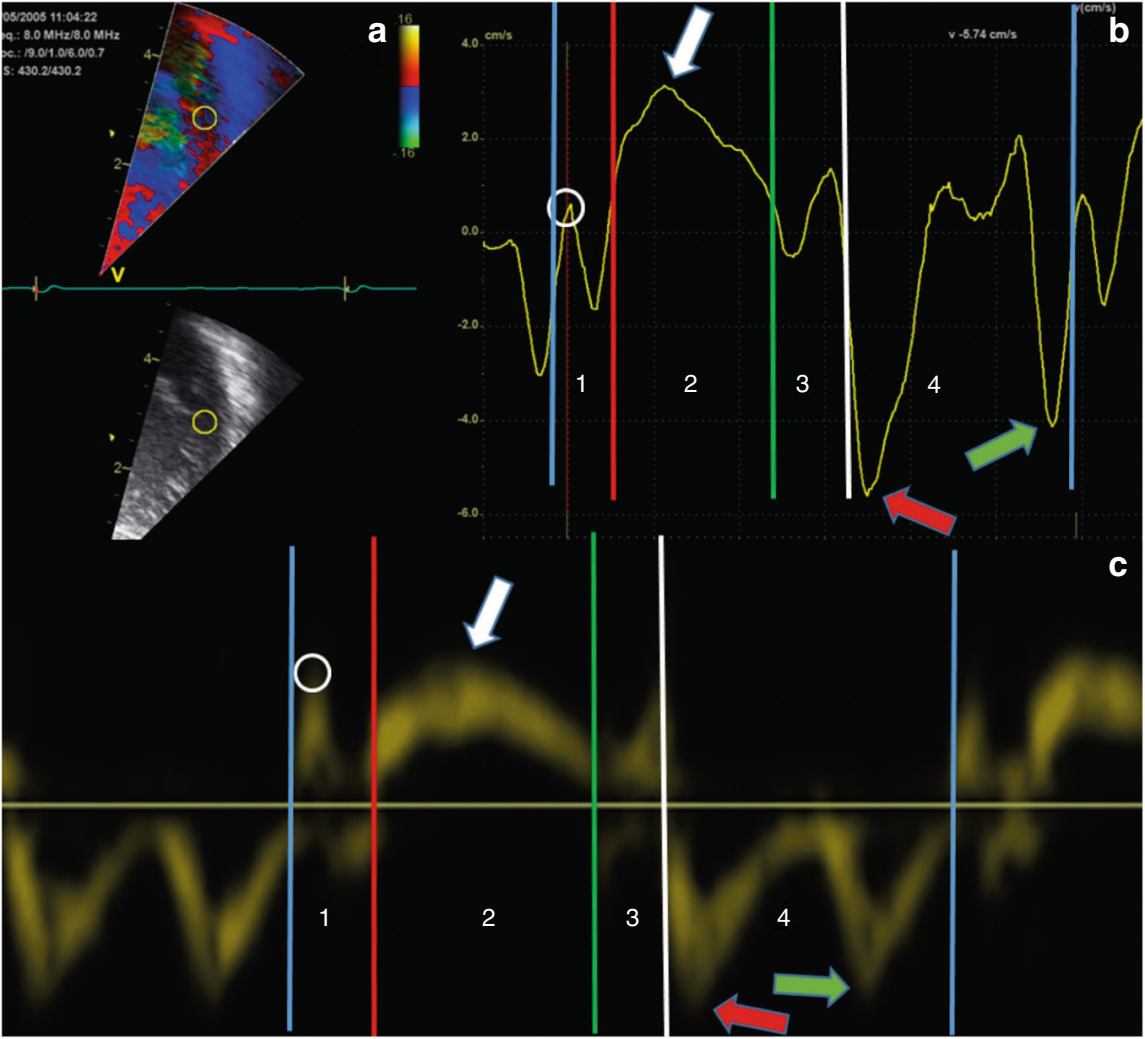
The upper panels show color-coded tissue Doppler velocity curve from a term neonate, from the left lateral atrioventricular plane from the apical four-chamber view. The upper left panel **a** shows the echocardiographic image with a sample area of 3 mm diameter (yellow circle). The curve in the upper right panel **b** shows the tissue velocities from the sample area. The lower panel **c** shows pulsed-wave tissue Doppler velocity curve from the septal hinge atrioventricular plane from the apical four-chamber view of a term neonate. White arrows at the peak systolic velocity (s′), red arrows at the early diastolic velocity (e′), and green arrows at the peak velocity in atrial systole (a′). The vertical lines denote the events defining the four phases of the heart cycle; isovolumic contraction,^1^ ejection phase,^2^ isovolumic relaxation,^3^ and filling.^4^ Note that the velocity curves in the isovolumic phases (1 and 3) contain one negative peak adjacent to the ejection phase^2^ and one positive peak adjacent to the filling phase,^4^ most evident in the color-coded measurements. Blue vertical lines denote closure of the mitral valve. Red vertical line denotes opening of the aortic valve. Green vertical line denotes closure of the aortic valve. White vertical line denotes opening of the mitral valve. White circles denote the peak isovolumic contraction velocity. *X*-axis: time. *Y*-axis: tissue velocity from the sample area

**Fig. 3:**
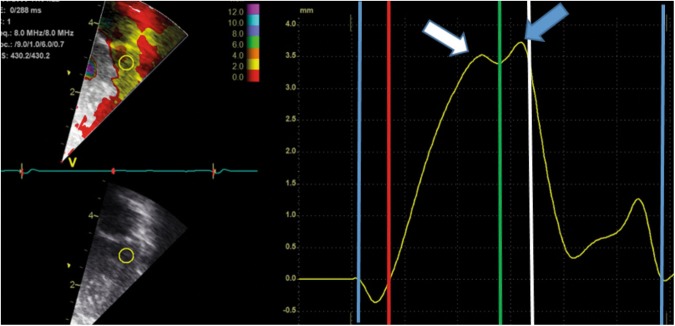
Same color-coded tissue Doppler velocity recording as in upper panel of Fig. 2, the curve now shows the displacement of the sample area. White arrow at the peak systolic displacement. Blue arrow at the peak displacement, occurring early in diastole in this image. Peak (global) displacement can also occur in systole. Blue vertical lines denote closure of the mitral valve. Red vertical line denotes opening of the aortic valve. Green vertical line denotes closure of the aortic valve. White vertical line denotes opening of the mitral valve. *X*-axis: time. *Y*-axis: displacement assessed by velocities from the sample area

There are two basic TDI techniques, pulsed-wave TDI (pwTDI) and color-coded TDI (cTDI). The user can use velocity–time curves by pwTDI (Fig. 1) and cTDI (Fig. 2, upper panel) to measure peak velocities and time intervals. In addition, the user can use cTDI to assess the displacement of the sample area throughout the cardiac cycle (Fig. 3).

The closure and opening of the ventriculo-arterial valves and the AV-valves define four mechanical phases of the cardiac cycle, the isovolumic contraction, ejection, isovolumic relaxation and filling phases (Figs. 2 and 3). The peak systolic velocity (s′) is the peak velocity in the ejection phase. There are two peaks in the filling phase, an early (e′) peak during the passive filling shortly after opening of the AV-valve and a late peak during the active filling in atrial systole (a′). By integrating the cTDI velocities with time, it is possible to assess displacement throughout the cardiac cycle. The closure of the aortic valve causes a notch in the displacement curve^10^ and the curve usually shows the peak systolic and peak global displacement on each side of this notch (Fig. 3). The peak global displacement occurs in systole if the peak in systole is higher than the peak in diastole. Several studies have assessed s′, e′, and a′ velocities and displacement in neonates. Other velocities derived measures are the peak velocity (Fig. 2, white circle) and the peak acceleration during the isovolumic contraction, the latter assessed as the positive slope of the velocity curve from baseline to the positive peak in the isovolumic contraction phase. These velocity measures have also been assessed in neonates,^11,12^ but less frequently than the s′, e′, and a′. The time resolution is important because a low frame-rate might lead to under-sampling of velocities^13^ and inaccurate assessments of time intervals,^14^ in particular for diastolic velocities and isovolumic phases of the cardiac cycle.

In general, measurements from right and left lateral AV-plane assess right and left cardiac function. Septal measurements are influenced by left- and right cardiac function, especially during the early transition phase of circulation. Studies in adults often average measurements (in particular displacement estimates) from the septal and left lateral wall for left ventricle assessment.^15^ Measurements in adults are similar but more accurate if averaged from apical four-chamber and two-chamber views.^16^ Studies have not yet compared these strategies in neonates; neonatal studies have so far only used apical four-chamber views and assessed measurements separately from the left lateral, septal and right lateral walls. Due to the elevated pulmonary pressures during the early neonatal period, the septum can be influenced by both left and right physiology, and therefore, it is preferable to report septal velocities separately.

The most commonly used time index by TDI is the myocardial performance index (MPI), also known as Tei index. Originally defined by time intervals from blood velocities,^17^ subsequent studies often define the index by time intervals from TDI velocity curves.^18^ The MPI is the sum of isovolumic phases as a fraction of the ejection phase (time interval 1 + 3 divided by time interval 2 in Fig. 2), usually assessed from the left lateral and right lateral hinge of the AV-plane. Other time indices studied are the electromechanical interval, assessed as the time from onset of QRS to the peak systolic velocity^12,19^ and indices related to duration of the isovolumic phases.^11,12^

**Fig. 4:**
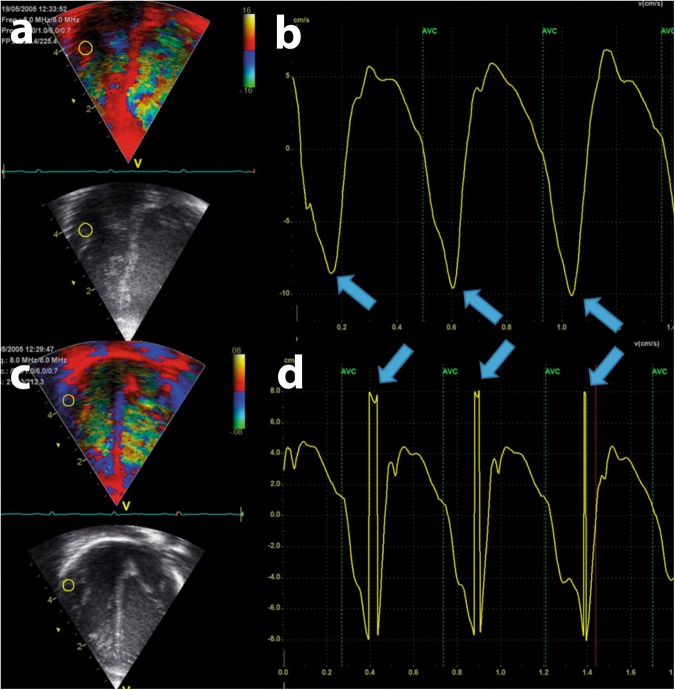
Example of non-aliased and aliased velocity measurements. Both panels show a color tissue Doppler velocity measurements of the right lateral atrioventricular plane in three consecutive heartbeats in a term neonate. The left part of both panels **a** and **c** show an apical four-chamber image with a sample area (yellow circle) of 3 × 3 mm. The right panels **b** and **d** show the velocity curves. The velocity scale is ±0.16 m/s in the upper panel and ±0.08 m/s in the lower panel. The negative peak velocity in diastole is approximately −0.09 m/s (blue arrows), within the velocity range in the upper panel but outside the velocity range in the lower panel. The ultrasound system therefore shows the velocity correctly in the upper panel and erroneously as a positive velocity close to the upper velocity limit in the lower panel. Note that there is complete fusion of diastolic velocities in the last two heart-cycles in the upper panel; it is not possible to identify two diastolic velocity peaks. *X*-axis: time. *Y*-axis: tissue velocity from the sample area

The velocity measurements of myocardium share many properties and limitations with measurements of blood velocities, including the Nyquist limit of velocity scale (the risk of aliasing velocities), limitations related to frame rate (time resolution) and angle dependency. Aliased velocity estimates occur when the muscle movement velocities are higher than the velocity scale. Figure 4 shows an example of normal and aliased velocities. Myocardial velocities seldom meet the “range × velocity product” limitation for velocity measurements even for high-frequency probes, and aliased tissue velocities usually results from inappropriate settings of velocity range (pulse repetition frequency, PRF) during acquisition rather than from the ultrasounds physical limitations due to probe frequencies and distance between probe and sample area.

## Measurements by pwTDI

**Fig. 5:**
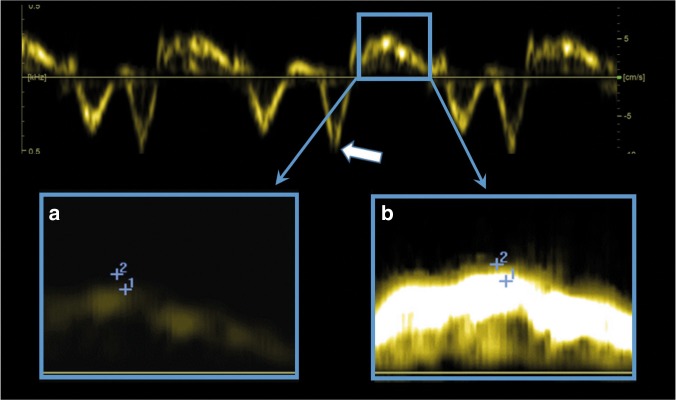
Pitfalls in image acquisition and analysis of pulsed-wave tissue Doppler images. The upper panel shows small breaks in the spectral curve when the ultrasound system records 2D images and pulsed-wave tissue Doppler spectral curve simultaneously. The a′ is false low in the second of the three heartbeats because the ultrasound system recorded the 2D image instead of the spectral curve at the time-point for the late diastolic peak (white arrow). The lower panel shows the systolic phase of one heart cycle with assessment of s′ velocity by correct (**a**) and by too high (**b**) gain setting. Measurement #1 is the correct velocity (5.1 cm/s) and measurement #2 is a false high velocity (6.1 cm/s). The only difference between the lower left and right panels is the gain setting during the off-line analysis

To obtain pwTDI velocities, the echocardiographer place a sample area stationary in the ultrasound sector and obtain a spectral image showing the myocardial velocities from this region by time (Fig. 1). Some ultrasound systems show the velocity measurements and the motion of the two-dimensional (2D) image simultaneously. The ultrasound system obtains the simultaneous 2D images and velocity curves by switching between the two modalities, causing small discontinuations in the spectral velocity curves (Fig. 5, upper panel). Measurements will be under-estimated if these breaks occur at the curve peaks. Therefore, for accurate measurements of velocities, the 2D image should be stopped to avoid these limitations. The ultrasound probe assesses velocities by emitting and receiving beams at a specified rate, referred to as the pulse repetition frequency (PRF). The PRF can typically be 1 kHz in neonates. Importantly, the PRF is not equivalent to frame-rate; to show the spectral velocity–time curves, the ultrasound system converts echo signals into velocity measurements by Fourier analyses and this procedure lowers the time resolution (equivalent to frame-rate). The relationship between PRF and time resolution (frame-rate) of the spectral curve is non-linear. By use of a phase-array probe transmitting at 2.4 MHz and a pulse repetition frequency of 1 kHz, the time resolution of the spectral curve can typically be 120 Hz and the velocity scale ±0.16 m/s. The effective time resolution is hence similar in pwTDI and cTDI and can often be higher in cTDI. The pwTDI spectral curves show the spectrum of velocities obtained from the region between the two calipers. In images with an inferior signal to noise ratio the band will be broader and contain more falsely low-velocity measurements, because velocity measurements of noisy signals will tend to be lower. As for blood velocities,^20^ the outer edge of the curves will show higher velocities if the gain setting during recording is set high, but no studies to date have explored the effect systematically in neonates.

It is customary to place the stationary sample area within the myocardial wall just apical to the AV-plane at end-diastole^15,21,22^ (Fig. 1) and use a low gain setting to avoid over-saturation of the curves. As the sample area is stationary in the ultrasound sector and the AV-plane moves throughout the cardiac cycle, the spectral curve will in most parts of the cardiac cycle display velocities of myocardium proximal or distal to the AV-plane rather than the true AV-plane velocities. While the width and the out-of-plane depth of the sample area are fixed, the distance between the two calipers (defining the length of the sample area) can be set at acquisition, applying variable degrees of spatial smoothing. A large distance between calipers will show velocities obtained from a larger area than a curve using a short distance between the calipers. Studies in neonates have often used a distance of 1–3 mm (some up to 5 mm)^23^ but no study has yet explored the impact from distance between calipers on measurements or repeatability.

After ensuring a sufficiently narrow angle of insonation (<20^o^),^24^ the echocardiographer records the spectral curves. The velocities can be measured either on the machine directly or offline. It is customary to lower the gain of the spectral curve to remove out-of-band noise and then measure the values at the peak of the curve band (Fig. 5, lower panel).^25^ The average velocity will be within the band and the outer edge of the band represents false-high velocities, explaining some of the systematic differences between pwTDI and cTDI measurements.^26^ High gain settings during recording and analysis increase the width of the band and hence the pwTDI values, and therefore give larger differences between pwTDI and cTDI measurements.^27^

## Measurements by cTDI

**Fig. 6:**
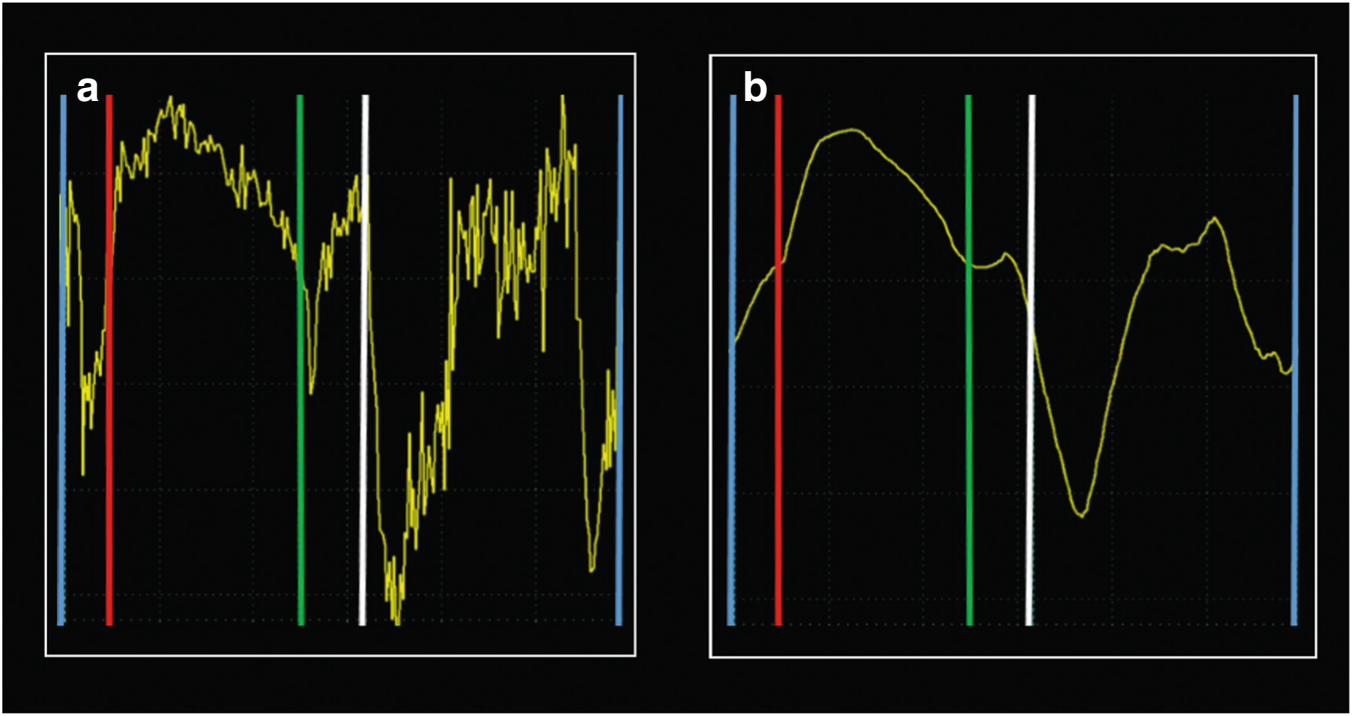
Same loop as in upper panel of Fig. 2, color-coded tissue Doppler velocity curves from a term neonate from the left lateral atrioventricular plane from the apical four-chamber view. The left curve (**a**) shows the velocities without time smoothing and the right curve (**b**) shows the velocities with too much time smoothing. Too much and too little time smoothing make interpretation of the curves difficult. Note that it is hard to identify the isovolumic contraction phase, especially in the right panel where the negative slope prior to the ejection phase is missing. Blue vertical lines denote closure of the mitral valve. Red vertical line denotes opening of the aortic valve. Green vertical line denotes closure of the aortic valve. White vertical line denotes opening of the mitral valve

When recording the cTDI images, the ultrasound system estimates velocities for each pixel in the TDI sector in each frame. The number of beams per frame is the main determinant of frame-rate. The frame-rate is high in narrow sectors because narrow sectors contain few beams. The frame-rate will be lower in deep sector than in shallow sectors, but the sector width (angle) has higher impact on frame-rate than sector depth. The user can increase frame-rate in a fixed-sized sector by reducing the beam density and hence reduce the lateral resolution. The output shows a color-coded velocity sector super-imposed on a gray-scale image with low frame-rate, similar to Doppler color flow mapping of blood. At a cTDI frame-rate of 200 Hz and using a probe transmitting at 5 MHz and a velocity scale of ±0.16 m/s, the cTDI sector will typically contain 40 beams, and if the echocardiographer increases the frame-rate to 400 Hz, the sector will typically contain 20 beams. During off-line analyses, the software displays velocities from sample areas within the cTDI sector as velocity–time curves, similar to pwTDI velocities. Importantly, the curve will show the average velocity from all pixels within the sample area, more resembling a curve within the middle of the pwTDI band than the edge of the band usually assessed by pwTDI. cTDI measurements enables a more extensive use of spatial averaging than pwTDI. The echocardiographer can adjust the level of averaging between adjacent pixels laterally and radially in the cTDI sector during recording and the analyzer can adjust the length and width of the sample area during analysis. Averaging will cause spatial smoothing due to more velocities being used in the measurements, but at the risk of including pixels with false velocities from stationary echoes (pericardium and stationary artefacts), fast-moving structures (valve leaflets), and velocities from areas other than the AV plane. In contrast to pwTDI, cTDI enables use of time-based averaging, both within a heartbeat by averaging consecutive velocity measurements and between heartbeats by averaging velocities assessed at the same time point in consecutive cardiac cycles. Averaging between consecutive heartbeats requires correct identification of the onset of heartbeats. Deformation imaging guidelines in adults recommend using the peak of the QRS complex as start of systole.^28^ Averaging techniques reduce errors from random noise but introduce systematic errors and reduce the effective time resolution.^27^ The use of averaging will always be a trade-off between these factors. Figure 6 shows the effects of time averaging on the velocity curves. Notably, too much time averaging could cause the isovolumic phases and isovolumic velocities to disappear from the velocity curves. The use of averaging in neonatal TDI assessment awaits further clarification.

cTDI requires a more comprehensive post-processing analysis to obtain measurements compared to pwTDI, but it has the advantage of the ability to assess the velocities from several areas simultaneously and more easily avoid areas with artefacts and poor image quality. cTDI enables stationary sample areas in the ultrasound sector or non-stationary sample areas within the sector to sample the tissue velocities from the AV valve plane throughout the cardiac cycle. Non-stationary sample areas improve repeatability and show velocity measurements different from stationary sample areas in adults.^29^ In addition to the averaging techniques applicable to cTDI velocity measurement, drift compensation is an additional averaging principle for cTDI displacement assessment.

## Differences between pwTDI and cTDI measurements, repeatability, and inter-vendor variations

### pwTDI versus cTDI measurements

The differences between pwTDI and cTDI prevent the measurements from being interchangeable. cTDI enables the visualization of multiple heart segments from one single view and measures mean systolic and diastolic velocities (rather than peak as is the case for pwTDI). As a result, velocities obtained using cTDI are generally 20% lower in systole and diastole compared to pwTDI. The two methods are therefore not interchangeable.^27^ The differences increase if large amounts of gain are applied to the pwTDI curves.^27^ pwTDI was introduced before cTDI, and more studies have used pwTDI than cTDI in adults and in neonates. Velocities by cTDI more closely resemble the true velocities in experimental settings.^26^ pwTDI velocities from within-the-band show better agreement against cTDI velocities than pwTDI velocities from the outer edge of the band.^26,30,31^ Similarly, displacements by integration of within-band pwTDI velocities show better agreement toward displacement by cTDI velocities and toward excursion by M-mode than integration of velocities from the outer edge of the band, both in phantom models and in-vivo analyses.^26,30,31^

### Repeatability

Most repeatability indices assess random variation. Repeatability will therefore be better in analysis of images with large amount of averaging applied. However, averaging will tend to lower peak velocity measurements^27^ and could therefore remove differences between the pathophysiological and normal clinical states. Studies report different indices for repeatability, with their strengths and weaknesses, and the user must interpret repeatability indices in relation to the population studied.

Few studies in neonates report test–retest repeatability. Most neonatal studies have assessed pwTDI repeatability by repeated assessment of velocity peaks and time intervals using the same spectral curve. cTDI recordings often show worse repeatability than re-assessment of pwTDI spectral curves, because cTDI repeatability tests usually includes position of a new sample area,^11,12,16^ the latter more resembling a test–retest situation. Typical coefficient of variation (COV) values range have been reported as 30–60% for isovolumic acceleration and 5–20% for cTDI velocities, displacements and time intervals,^12^ while authors report COVs as low as 4–8 % for pwTD velocities.^32^

### Inter-vendor variation

Inter-vendor variation has gained much attention for newer imaging modalities, but the inter-vendor agreement for tissue Doppler indices seems better than for conventional measurements of blood velocities and dimensions.^33^ Vendors have put much effort into standardizing measurements and inter-vendor variation was probably a more significant obstacle in the past. Authors have found good agreement for measurements between vendors in a recent study^26^ while earlier studies have found systematic differences between vendors for pwTDI^34^ and cTDI^35^ measurements, with bias up to 25% and 15%, respectively.

## Interpretation of tissue Doppler velocities and displacement values

In general, large peak systolic AV-plane velocities, accelerations, and displacements imply better myocardial function. This can be a result of better contractility or favorable loading conditions. Large hearts have higher velocities and displacements than small hearts.^36,37^ To compare between hearts of different sizes, the neonatologist can normalize measurements for cardiac size. Neonatal studies have often used end-diastolic septal length or length from apex to the lateral AV-plane hinge for normalization for size^38,39^ and reported velocities and excursions normalized by LV length in neonates and children.^38–40^ Others have suggested normalizing by dividing the velocities by the end-diastolic diameter of the LV.^19^ By using the end-diastolic ventricle length as a measure for cardiac size, peak systolic velocity (s′) adjusted for cardiac size (i.e., divided by end-diastolic ventricle length) will express the peak systolic strain rate for the region between apex and the AV-valve plane, and, similarly, displacement adjusted for cardiac size will express the peak strain for the region. We therefore suggest end-diastolic length of the ventricle wall as the most suitable index for normalization for size, assessed between the echo from the pericardium at the apex and the septal hinge of the mitral wall.

Preload and afterload have impact on systolic velocities and deformation indices.^41^ As loading conditions have less impact on peak systolic strain rate than peak systolic strain,^8^ loading conditions possibly have less impact on systolic velocities than on displacements. Changes in peak systolic velocities (when adjusted for heart size or compared between hearts of similar size) can imply changes in contractility, but importantly, the neonatologist must consider the loading condition when interpreting the values. Higher diastolic velocities can imply better myocardial relaxing function, but interpretation of diastolic velocities is more challenging because more factors influence the measurement. Restoring forces from the systolic contraction, active relaxation (diastolic function), load (venous pressure), and atrial contraction could influence diastolic velocity measurements.

In neonates, the e′ and a′ peaks tend to fuse at higher heart rates. The a′ is the peak during atrial systole. If the atrial systole occurs shortly after the onset of diastole, the a′ velocity wave could “climb” on top of the e′. The velocity curve could then show only one diastolic peak, as shown in the two last cardiac cycles in the upper panel of Fig. 4. Studies have approached this challenge differently, some by reporting only the largest diastolic velocity^19,38^ and others by reporting one diastolic peak in case of fusion and two peaks if two diastolic peaks could be separated.^12^

The peak E mitral blood flow velocity divided by peak e′ tissue Doppler velocity (E/e′) has been assessed in neonates.^42,43^ In adults with heart failure with preserved ejection fraction, E/e′ is used as an index of diastolic heart function.^15^ A large E/e′ could indicate a high filling pressure and poor diastolic function. Guidelines in adults recommend use of pwTDI rather than cTDI velocities in estimation of E/e′ because the tissue Doppler velocities vary between the modalities and more validation studies have used pwTDI than cTDI.^15^ Its role as a function index in neonates awaits further clarification.

## Interpretation of time indices

Indices based on time intervals are independent of heart size and the angle of insonation, making comparisons between different heart sizes and during growth easier. The first publication of MPI (Tei index) proposed this index as a relatively load-independent measure of contractility^17^ by use of the time intervals from blood-Doppler velocity curves. Although studies have found significant associations between MPI and several pathological states, authors have questioned the use of MPI as an index of contractility, finding the index highly dependent on load and even questioned its ability to assess changes in contractility.^44^ Several studies have reported MPI by tissue Doppler in neonates.^6,11,42,43,45–47^ A low value might reflect a better cardiac function than a high value, but the neonatologist must consider loading conditions when interpreting MPI, as MPI increases with increased afterload and with reduced preload. Its use in neonates awaits further clarification. Neonatal studies have also assessed the isovolumic time intervals.^11,12,19,48^ Short isovolumic phases generally imply better cardiac function, but several factors influence time interval measurements and make their use as cardiac function indices challenging. The time interval from onset of QRS to peak s′ velocity has in experimental settings been regarded as an index of maturation of the electromechanical coupling, with a short time interval showing a more mature coupling, thus implying a better cardiac function. Recently, Kahr and colleagues showed that the time interval decreases with postnatal age in neonates.^19^

## Assessment in neonates

### Image acquisition

Table 1 shows important factors during acquisition and analysis of tissue Doppler velocities and time intervals. Apical four-chamber view is the most used view for velocity measurements. It is important to position the probe at the apex and have good signals from the region of interest (AV-valve plane). For pwTDI velocities, we recommend placing the stationary sample area just apical to the AV plane at end-diastole, acknowledging that the sample area will obtain velocities from atrial tissue at end-systole and velocities from ventricle myocardium at end-diastole. An angle of insonation less than 20° needs to be maintained to avoid underestimating velocities. A velocity range ±0.16 m/s will avoid aliasing in most cases in neonates. We suggest using a low gain setting to avoid oversaturated spectral images and freezing the 2D image during spectral image acquisition before recording several cardiac cycles. We suggest using a sample area (distance between the calipers) of 2–3 mm, acknowledging that no studies have assessed the effect of different sizes for the sample area in neonates.

**Table 1:** Suggestions and tips for obtaining pwTDI and cTDI velocities and time indices

**A. General**
1. Obtain a good apical position. Use apical four-chamber view
2. Adjust velocity scale to avoid aliasing (±0.16 m/s)
3. Record several heartbeats
4. Use ECG recording on the ultrasound system to separate between a′ and the negative peak in the isovolumic contraction phase
5. Average measurements over several heartbeats
6. Peak values by pwTDI and cTDI are not interchangeable
7. Use the same technique and settings for longitudinal assessment
**B. Indices by pulsed-wave tissue Doppler**
1. Freeze the 2D image when obtaining the spectral curve
2. Avoid high gain setting
3. Place sample area just apical to the AV plane at end-diastole
4. Use a sample area (distance between the two calipers) of 2–3 mm
5. Use low gain settings in off-line analyses
**C. Indices by color-coded tissue Doppler**
1. Use a narrow and shallow sector to ensure high frame-rate
2. Check the image quality
3. Adjust position and size of sample area to avoid errors and obtain a good-quality curve
4. Use a sample area of 2 × 2 mm (premature) or 3 × 3 mm (mature)
5. Use averaging techniques with care; although they improve repeatability they can be too powerful and might mask true events

In the cTDI analyses, frame-rate will have impact on the time resolution of measurements. A high frame-rate will reduce the risk of missing true peak values between frames. A small sector, especially a narrow sector, will enable high frame-rates while maintaining a good lateral spatial resolution. If a larger sector is used, it is possible to obtain movement from right, septal, and left hinge points of the AV plane in a single recording. This reduces the burden of repeated image acquisition and enables the assessment of left- and right-cardiac function from the same image. However, a large sector will reduce the time resolution (frame-rate), the lateral spatial resolution (few beams in the sector), or both. The frame-rate requirements are probably more critical for short-time intervals and for diastolic velocities than for long-time intervals, systolic velocities, and displacements.^13,14,49^ The sector should be narrow and shallow to keep the frame-rate high, but it must show the relevant structures throughout the cardiac cycle. Acquisition of several cardiac cycles will enable direct comparisons between heartbeats and is a prerequisite for averaging between heartbeats.

### Assessment of peak velocities and displacement

We suggest defining onset of systole at the peak of the QRS complex, in accordance with deformation imaging guidelines in adults.^28^ The pwTDI gain should be low to avoid overestimating pwTDI velocities. For obtaining the cTDI curves during off-line analysis, studies have used different sizes for the sample area. We suggest a sample area of 2 × 2 mm in premature and 3 × 3 mm in term neonates. The analyses should use averaging techniques with caution, especially for short timing intervals and diastolic peaks. Studies should describe in detail the use of averaging and neonatologists should use similar averaging settings for longitudinal assessment in clinical practice. Between-beat averaging techniques require regular rhythm and proper ECG recordings to ensure that the user uses the same start point for each heartbeat. Tracing of the cTDI sample area to keep it at the AV hinge during the cardiac cycle has impact on the measurements and repeatability in adults^29^ but no study has yet systematically explored the difference between measurements from traced and stationary sample areas in neonates.

The systolic peak is the peak during the ejection phase. The peak in the left lateral wall occurs earlier in the ejection phase than the septal and right lateral peaks. If there are two distinct diastolic peaks in the filling phase and the velocity curve shows low velocities in mid-diastole, the peaks should be assessed as the e′ and a′ velocities. With an increasing degree of fusion, the interpretation of the two peaks as separate values becomes more difficult. If two diastolic peaks can be clearly identified, the first peak could probably be regarded as an e′. By increased degree of fusion, e′-velocities will have more impact on the second peak (the a′-wave will climb on top of the e′-wave) and interpretation of the second peak as a function index becomes more challenging. In case of complete fusion, the velocity curve will show only one diastolic peak. The a′-wave occurs simultaneously with the P-wave on the ECG-recording, while the isovolumic contraction occurs simultaneously with the QRS-wave. In cases of complete fusion of e′ and a′, the operator could therefore use the ECG signal to avoid interpreting the negative peak in the isovolumic contraction (Fig. 2) as an a-wave. Studies should report how they approach the challenge of fusion of diastolic velocities. Interpretation of diastolic velocities acquired in heartbeats with different degrees of velocity fusion could be difficult.

In the isovolumic contraction phase, the peak velocity is usually positive (white circle in Fig. 2). The isovolumic acceleration is the slope of the velocity curve from baseline to the positive peak velocity during the isovolumic phase (Fig. 2).

Measurements of displacements by cTDI assess motion distance relative to end-diastole. The user can define onset of systole either by mechanical properties (closure of the mitral valve or opening of the aortic valve) or by electrical properties (peak of the QRS complex). This will have an impact on the measurements and we recommend defining onset of systole at the peak of the QRS complex to standardize measurements. If the peak of the QRS complex defines the onset of systole, there will be an early-systolic displacement away from the probe (similar to systolic stretch by strain analysis) before onset of mechanical movement towards the apex. When applying between-beat averaging or drift compensation for cTDI displacement analyses, it is important to ensure that the curves for each heartbeat start at the same point in the cardiac cycle and that the cardiac rhythm is regular. The closure of the aortic valve causes a notch in the displacement curves, especially for the left lateral and septal walls (Fig. 3). Peak systolic displacement is the peak of the displacement curve prior to the notch, whereas the global displacement is the global peak of the curve; importantly, the systolic and global peaks are not interchangeable.^10^

### Assessment of time-derived indices

Although the angle of insonation is less important for time indices than for velocities and displacements, the sonographer should ensure a good angle of insonation because time indices will always need to be interpreted together with the velocities and displacements. In the velocity curves, the isovolumic contraction and relaxation phases each show a positive and a negative velocity peak, the negative peaks on each side of the ejection phase and the positive peaks on each side of the filling phase^14^ (Fig. 2). A high frame-rate is a prerequisite for obtaining short-time intervals correctly, and if the user uses large amounts of smoothing, the identification of the isovolumic phases becomes difficult (Fig. 6). Time indices from the left lateral and right lateral walls assess left and right cardiac function, respectively.

### Measurements in neonates and during transition

As for tissue Doppler velocity measurements in adults, neonatal studies often present pwTDI rather than cTDI. Studies of displacement have used cTDI and most studies of time intervals have used pwTDI. There are several studies describing reference ranges of tissue Doppler velocities and displacements from the apical four-chamber view in premature neonates^5,6,12,36,38,40,42,46,50–55^ and term neonates.^6,11,12,32,51,52,54,56,57^ Some are longitudinal studies with repeated measurements over days and weeks. Table 2 shows measurements by pwTDI and cTDI from some studies. In general, right cardiac velocities and displacement are higher than left cardiac indices. Measurements tend to increase over time, likely reflecting increasing heart size and improved function. Indices normalized for heart size tend to increase by maturation. Changes in right side measurements by maturation are often larger than changes in septal and left side measurements, possibly because changes in loading conditions have larger impact on right cardiac function. cTDI velocity measurements are lower than pwTDI measurements. Many studies in premature neonates show a transient decline in myocardial velocities during the first hours of life.^23^ In some studies, E/e′ seems relatively independent of growth, with typical values ranging 5–10.^42,46,51^

**Table 2:** Peak systolic (s′), early diastolic (e′), and late diastolic (a′) velocities and displacement of the AV-valve plane in premature and mature neonates. Pulsed-wave (pwTDI) and color-coded tissue Doppler (cTDI) indices (mean (standard deviation)). GA: Gestational age

Reference and TDI mode	Index	Location		
		Left lateral	Septum	Right lateral
Lee et al.^38^ cTDI				
GA < 28 weeks First 24 h	Systolic peak (s′) (cm/s)	1.8 (0.7)		2.8 (0.9)
	Diastolic peak (cm/s)	2.3 (1.1)		4.1 (1.6)
	Displacement (mm)	1.9 (0.9)		3.3 (1.2)
Joshi et al.^12^ cTDI				
Premature (≤34 weeks) and mature (≥38 weeks) Within first 72 h	Systolic peak (s′) (cm/s)	2.4 (0.9)	2.9 (0.9)	4.4 (1.1)
	Early diastolic peak (e′) (cm/s)	4.9 (2.4)	4.7 (2.3)	6.2 (2.7)
	Late diastolic peak (a′) (cm/s)	2.9 (1.0)	3.7 (1.1)	5.8 (1.3)
	Displacement (mm)	3.9 (1.7)	4.7 (1.2)	7.5 (1.9)
Saleemi et al.^53^ pwTDI				
GA 24–27 weeks 48 h of age	Systolic peak (s′) (cm/s)	3.5 (1.0)		
	Early diastolic peak (e′) (cm/s)	3.8 (0.9)		
	Late diastolic peak (a′) (cm/s)	4.3 (1.0)		
GA 28–31 weeks 48 h of age	Systolic peak (s′) (cm/s)	3.8 (0.8)		
	Early diastolic peak (e′) (cm/s)	3.9 (1.0)		
	Late diastolic peak (a′) (cm/s)	4.2 (0.7)		
GA 32–35 weeks 48 h of age	Systolic peak (s′) (cm/s)	4.7 (1.2)		
	Early diastolic peak (e′) (cm/s)	4.8 (1.2)		
	Late diastolic peak (a′) (cm/s)	5.2 (0.9)		
Breatnach et al.^55^				
GA < 29 weeks First 24 h (see paper for further time points)	Systolic peak (s′) (cm/s)	2.8 (0.9)	2.4 (0.6)	3.6 (0.9)
	Early diastolic peak (e′) (cm/s)	3.6 (1.4)	2.8 (0.8)	3.9 (1.3)
	Late diastolic peak (a′) (cm/s)	4.0 (1.5)	3.9 (1.1)	3.9 (1.1)
Ciccone et al.^54^ pwTDI				
GA 31–36 weeks Days 3–4	Systolic peak (s′) (cm/s)	5.4 (0.8)	4.5 (1.4)	5.5 (0.9)
	Early diastolic peak (e′) (cm/s)	7.6 (1.2)	2.7 (0.7)	6.2 (1.3)
	Late diastolic peak (a′) (cm/s)	7.5 (1.3)	6.5 (0.7)	9.4 (1.1)
GA 37–41 weeks Day 3–4	Systolic peak (s′) (cm/s)	5.6 (0.8)	5.1 (0.4)	6.1 (0.9)
	Early diastolic peak (e′) (cm/s)	7.9 (1.2)	6.1 (0.8)	7.9 (1.1)
	Late diastolic peak (a′) (cm/s)	7.5 (1.3)	6.7 (0.8)	9.5 (1.1)
Negrine et al.^51^ pwTDI				
GA < 30 weeks First 24 h	Systolic peak (s′) (cm/s)	3.7 (0.6)		5.0 (0.6)
	Early diastolic peak (e′) (cm/s)	4.2 (0.8)		4.2 (1.1)
	Late diastolic peak (a′) (cm/s)	5.4 (2.2)		7.3 (1.1)
GA 30–36 weeks First 24 h	Systolic peak (s′) (cm/s)	4.3 (0.7)		5.9 (0.9)
	Early diastolic peak (e′) (cm/s)	5.7 (1.4)		6.2 (1.0)
	Late diastolic peak (a′) (cm/s)	6.0 (1.9)		7.7 (1.8)
Term First 24 h	Systolic peak (s′) (cm/s)	5.3 (1.0)		6.9 (1.2)
	Early diastolic peak (e′) (cm/s)	6.4 (1.2)		7.3 (1.1)
	Late diastolic peak (a′) (cm/s)	7.1 (1.8)		8.1 (1.6)
Mori et al.^32^ pwTDI				
Term First 24 h	Systolic peak (s′) (cm/s)	5.3 (0.9)	3.7 (0.6)	6.2 (1.1)
	Early diastolic peak (e′) (cm/s)	7.5 (1.5)	5.0 (1.0)	7.5 (1.4)
	Late diastolic peak (a′) (cm/s)	6.2 (1.4)	4.9 (1.0)	9.2 (1.6)

Most studies of time intervals by tissue Doppler have used pwTDI.^5,6,11,12,19,43,45,48^ Typical MPI average values reported are 0.3-0.6, and MPI decreases with gestational age, with maturation and with reduced load. Average isovolumic contraction and relaxation time intervals reported are 40–80 ms, and isovolumic acceleration values are typically 0.5–1 m/s^2^. Time intervals from onset of QRS to the S′ reported are 95–115 ms. MPI values obtained using flow Doppler significantly differ to those obtained using TDI. This is expected as flow Doppler measures event timings based on flow of blood while pwTDI measures event timings based on movement of muscle tissue. The higher time resolution of TDI enables a more accurate assessment of the onset and cessation of the different events throughout the cardiac cycle.

## Examples from studies of pathological conditions in neonates

Tissue Doppler indices from apical four-chamber views often show differences between healthy and sick neonates. Observational studies comparing tissue Doppler indices against conventional indices often show better discriminating capabilities. Vitali and colleagues showed that surfactant administration to premature neonates with respiratory distress syndrome improved right cardiac systolic velocities 2 h after the procedure.^58^ In anemia of the premature, pwTDI velocities 3 days after transfusion of red blood cells were higher than prior to transfusion without changes in shortening fraction.^59^ In perinatal asphyxia, tissue Doppler velocities and time intervals were worse than in controls while fractional shortening was similar between groups.^3,60^ Analysis in septic neonates found worse tissue Doppler velocities and time indices than in controls while conventional indices showed no discriminating capabilities.^4^ MPI was a significant marker of survival in the septic neonates in the same study. In neonates with persistent high pulmonary vascular resistance, right lateral s′, right lateral e′ and septal e′ pwTDI velocities were worse than in controls, while septal s′ and right lateral a′ and septal a′ were similar.^61^ Several studies have assessed tissue Doppler indices in bronchopulmonary dysplasia (BPD). Yates and colleagues found worse left MPI and left and right E/e′ in BPD neonates but similar tissue Doppler velocities and right MPI compared to controls.^62^ Sehgal and colleagues found worse RV peak systolic and isovolumic velocities and E/e′ and MPI compared to premature controls at 36 weeks, and that E/e′ was associated with duration of subsequent respiratory support.^43^ Czernic and colleagues found similar initial right side MPI in premature neonates with and without subsequent development of BPD, and that MPI remained high in those developing BPD.^47^ Recently, Yajamanyam and colleagues found worse right, septal, and left MPI, higher right s′ velocity and right isovolumic relaxation time in premature neonates with severe and with mild BPD compared to premature neonates with no BPD, while the other tissue Doppler velocities were similar between groups.^63^ The latter study could indicate a compensatory increase in right cardiac contractility to overcome worse right cardiac afterload. Acute changes in load occur in closure of a patent ductus arteriosus (PDA). El-Khuffash and colleagues showed worse left, septal, and right tissue Doppler velocities 1 and 18 h after surgical closure of a PDA and higher (worse) time indices from the left lateral wall at both time points.^64^ Parikh and colleagues found worse left and right tissue Doppler velocities and MPI in premature neonates with hemodynamically significant PDA, and patients whose PDA remained open despite indomethacin had worse velocities and MPI compared to those with successful closure by indomethacin.^65^ Several studies have assessed tissue Doppler velocities and time intervals in neonates born small for gestational age.^45,48,66–68^ These show differences in tissue Doppler velocities and MPI not detected by conventional indices of cardiac function.

## Conclusion

Use of indices derived from tissue Doppler velocity–time curves in neonates is feasible. They detect differences between neonates in normal and pathological conditions not diagnosed by conventional indices. Systolic velocities and displacements are well-established indices of cardiac function in neonates, while interpretation of tissue Doppler time intervals and diastolic velocities as markers of cardiac function is more challenging. Standardization of acquisition and interpretation of indices will facilitate its use as a bedside tool in neonatal intensive care. Although less dependent on loading conditions than conventional indices, these new indices cannot assess the contractility per se. The neonatologist must take loading conditions and the interplay between the right and left side into account when interpreting measurements. In general, high preload and low afterload are associated with higher velocity and acceleration indices and shorter time intervals. As for most diagnostic tools, studies have not yet shown that medical interventions based on abnormal TDI measurements lead to improvements in patient outcomes. Interventions should be based on evaluation of the combination of clinical state and available cardiac function indices, and not be solely based on one parameter. Further research steps should focus on exploring the impact of pathophysiologic processes in neonatal hearts on these new indices, especially on how and when to apply therapeutic interventions guided by tissue Doppler indices. In the interim, we recommend that, when feasible, tissue Doppler indices of velocity and event timings should form part of a routine functional echocardiogram in the neonatal population.

## APPENDIX

**European Special Interest Group ‘Neonatologist Performed Echocardiography’ (NPE), endorsed by the European Society for Paediatric Research (ESPR) and European Board of Neonatology (EBN)**

**de Boode W. P.** (chairman), Department of Neonatology, Radboud University Medical Center, Radboud Institute for Health Sciences, Amalia Children’s Hospital, Nijmegen, The Netherlands (willem.deboode@radboudumc.nl)

**Austin T.**, Department of Neonatology, Rosie Hospital, Cambridge University Hospitals NHS Foundation Trust, Cambridge, United Kingdom (topun.austin@addenbrookes.nhs.uk)

**Bohlin K.**, Department of Neonatology, Karolinska University Hospital, Karolinska Institutet, Stockholm, Sweden (kajsa.bohlin@ki.se)

**Bravo M. C.**, Department of Neonatology, La Paz University Hospital, Madrid, Spain (mcarmen.bravo@salud.madrid.org)

**Breatnach C. R.**, Department of Neonatology, The Rotunda Hospital, Dublin, Ireland (colm.breatnach@gmail.com)

**Breindahl M.**, Karolinska University Hospital, Karolinska Institutet, Stockholm, Sweden (morten.breindahl@sll.se)

**Dempsey E.**, INFANT Centre, Cork University Maternity Hospital, University College Cork, Ireland (g.dempsey@ucc.ie)

**El-Khuffash A.**, Department of Neonatology, The Rotunda Hospital, Dublin, Ireland; Department of Pediatrics, The Royal College of Surgeons in Ireland, Dublin, Ireland (afifelkhuffash@rcsi.ie)

**Groves A. M.**, Division of Newborn Medicine, Mount Sinai Kravis Children’s Hospital, New York, NY, USA (alan.groves@mssm.edu)

**Gupta S.**, University Hospital of North Tees, Durham University, Stockton-on-Tees, United Kingdom (samir.gupta@nth.nhs.uk)

**Horsberg Eriksen B.**, Department of Pediatrics, Møre and Romsdal Hospital Trust, Ålesund, Norway (beate.eriksen@me.com)

**Levy P. T.**, Department of Pediatrics, Washington University School of Medicine, Saint Louis, MO, USA; Department of Pediatrics, Goryeb Children’s Hospital, Morristown, NJ, USA (Levy_P@kids.wustl.edu)

**McNamara P. J.**, Departments of Pediatrics and Physiology, University of Toronto, Toronto, ON, Canada (patrick.mcnamara@sickkids.ca)

**Molnar Z.**, John Radcliffe Hospital, Oxford, United Kingdom (zoltan.Molnar@ouh.nhs.uk)

**Nestaas E.**, Institute of Clinical Medicine, Faculty of Medicine, University of Oslo, Norway; Department of Cardiology and Center for Cardiological Innovation, Oslo University Hospital, Rikshospitalet, Oslo, Norway; Department of Paediatrics, Vestfold Hospital Trust, Tønsberg, Norway (nestaas@hotmail.com)

**Rogerson S. R.**, The Royal Women's Hospital, Parkville, VIC, Australia (sheryle.Rogerson@thewomens.org.au)

**Roehr C. C.**, Department of Paediatrics, University of Oxford, John Radcliffe Hospital, Oxford, United Kingdom (charles.roehr@paediatrics.ox.ac.uk)

**Savoia M.**, Azienda Ospedaliero-Universitaria S. Maria della Misericordia, Udine, Italy (marilena.savoia@gmail.com)

**Schubert U.**, Department of Clinical Science, Intervention and Technology, Karolinska Institutet, Stockholm, Sweden (ulfschubert@gmx.de)

**Schwarz C. E.**, Department of Neonatology, University Children’s Hospital of Tübingen, Tübingen, Germany (c.schwarz@med.uni-tuebingen.de)

**Sehgal A.**, Department of Pediatrics, Monash University, Melbourne, Australia (arvind.sehgal@monash.edu)

**Singh Y.**, Addenbrooke's Hospital, Cambridge University Hospitals NHS Foundation Trust, Cambridge, United Kingdom (yogen.Singh@nhs.net)

**Slieker M. G.**, Department of Paediatric Cardiology, Radboudumc Amalia Children’s Hospital, Nijmegen, The Netherlands (Martijn.Slieker@radboudumc.nl)

**Tissot C.**, Department of Pediatrics, Clinique des Grangettes, Chêne Bougeries, Switzerland (cecile.tissot@hotmail.com)

**van der Lee R.**, Department of Neonatology, Radboud University Medical Center, Radboud Institute for Health Sciences, Amalia Children’s Hospital, Nijmegen, The Netherlands (Robin.vanderLee@radboudumc.nl)

**van Laere D.**, Department of Pediatrics, Antwerp University Hospital UZA, Edegem, Belgium (david.VanLaere@uza.be)

**van Overmeire B.**, Department of Neonatology, University Hospital Brussels, Brussels, Belgium (bart.van.overmeire@erasme.ulb.ac.be)

**van Wyk L.**, Department of Paediatrics & Child Health, University of Stellenbosch, Cape Town, South Africa (lizelle@sun.ac.za)
